# Perspectives for the Treatment of Brucellosis in the 21st Century: The Ioannina Recommendations

**DOI:** 10.1371/journal.pmed.0040317

**Published:** 2007-12-27

**Authors:** Javier Ariza, Mile Bosilkovski, Antonio Cascio, Juan D Colmenero, Michael J Corbel, Matthew E Falagas, Ziad A Memish, Mohammad Reza Hasanjani Roushan, Ethan Rubinstein, Nikolaos V Sipsas, Javier Solera, Edward J Young, Georgios Pappas

## Abstract

The authors provide evidence-based guidance on treating human brucellosis, and discuss the future clinical trials that would help address the controversies surrounding treatment.

Brucellosis is probably the commonest anthropozoonotic infection worldwide [1–3], but remains in various aspects an enigma in the 21st century [4]. Brucella melitensis remains the major cause of human disease worldwide, followed by B. abortus and B. suis, while rare but persisting cases of B. canis human infection and disease by novel Brucella pathogens of marine mammals have also emerged. The disease is re-emerging as a significant cause of travel-related disease [5] and represents an index of poor socioeconomic status (Figure 1). Its treatment is largely based even today on the principles applied half a century ago by pioneer researchers [6] and few modifications have been made in the following years, despite the emergence of new antibiotic classes and different therapeutic approaches [7].

**Figure 1:**
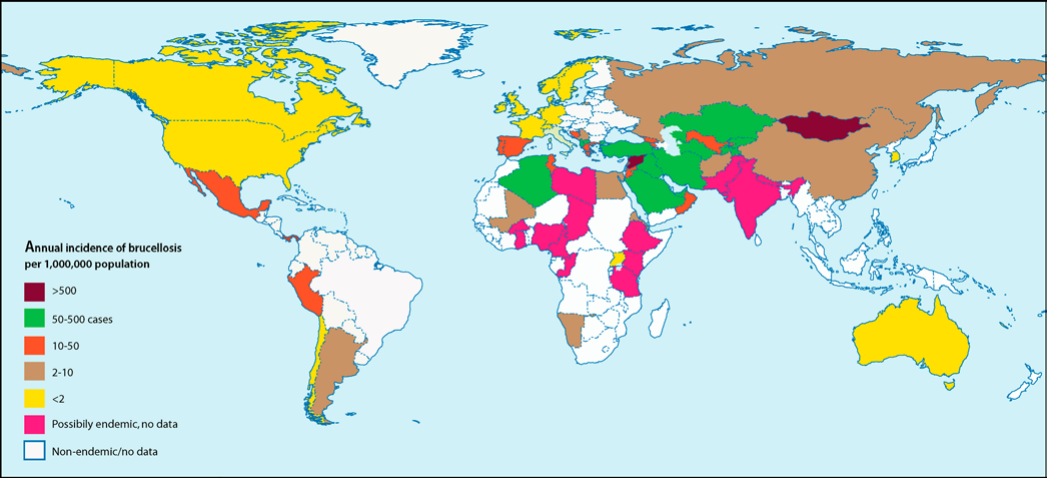
The Global Incidence of Human Brucellosis Reproduced from: Gutierrez Ruiz C, Miranda JJ, Pappas G (2006) A 26-year-old man with sternoclavicular arthritis. PLoS Med 3(8): e293. doi:10.1371/journal.pmed.0030293 Derived from: Pappas G, Papadimitriou P, Akritidis N, Christou L, Tsianos EV (2006) The new global map of human brucellosis. Lancet Infect Dis 6: 91-99.

The World Health Organization (WHO) issued recommendations for the treatment of human brucellosis in 1986 [8], suggesting the use of doxycycline, 100 mg twice daily for six weeks combined with either rifampicin, 600–900 mg daily for six weeks, or streptomycin, 1 g daily for 2–3 weeks. During the following years, a number of clinical studies assessed the efficacy of different regimens. Furthermore, reports from various regions of the world revealed that the WHO-recommended regimens have not been universally applied in clinical practice. More importantly, these regimens still allow for a small, albeit significant percentage of therapeutic failures, most commonly in the form of relapses, ranging from 5% to 15% of uncomplicated cases. Although these relapses are usually mild and can be treated successfully with the same regimens, they represent a significant morbidity factor. Risk factors for relapse have been assessed [9,10], but it remains unclear what is the best regimen to be used in their presence.

**Summary Points**

- Brucellosis remains the commonest anthropozoonosis worldwide, and its treatment remains complex, requiring protracted administration of more than one antibiotic.
- In November 2006, a consensus meeting aimed at reaching a common specialist statement on the treatment of brucellosis was held in Ioannina, Greece under the auspices of the International Society of Chemotherapy and the Institute of Continuing Medical Education of Ioannina.
- The author panel suggests that the optimal treatment of uncomplicated brucellosis should be based on a six-week regimen of doxycycline combined either with streptomycin for 2–3 weeks, or rifampicin for six weeks. Gentamicin may be considered an acceptable alternative to streptomycin, while all other regimens/combinations should be considered second-line.
- The development of a common global therapeutic language for human brucellosis, and future, properly conducted clinical trials would definitely solve controversies regarding the disease.

Another controversial subject in brucellosis treatment has been the duration of therapy: If the treatment is prolonged, the risk of relapse progressively decreases. Obviously, defining an acceptable relapse rate based on the duration of the schedule used is somewhat arbitrary.

In an era of rapid emergence of antimicrobial resistance, controversies regarding the prolonged use of antibiotics with established activity against Brucella pose special problems. In some endemic regions, especially in the developing world, brucellosis and tuberculosis coexist in the same communities. So for example, for those medications active against tuberculosis that are also used for the treatment of brucellosis, such as rifampicin, there is an increased potential in the community for emergence of mycobacterial resistance to these antimicrobials because of their use against brucellosis.. The public health significance of such resistance may be far larger than the cumulative brucellosis morbidity [11]. Finally, it should be kept in mind that the majority of human and animal brucellosis cases occur in resource-deprived countries of the developing world. Therefore, the cost of the new, sophisticated antimicrobials should be taken into account when designing an effective therapy for a large number of patients.

## Development of the “Ioannina Recommendations”

In November 2006, the 1st International Meeting on the Treatment of Human Brucellosis was held in Ioannina, Greece, co-organized by the Institute of Continuing Medical Education of Ioannina and the International Society of Chemotherapy, as part of the latter's Disease Management Series. The aims of the meeting were to: review the current situation of the disease worldwide; summarize the existing knowledge regarding the pathogenesis of risk factors for, and natural history of, brucellosis; review recent basic science and clinical developments in the field of diagnosis and treatment of brucellosis; and set the background for the production of a statement on the current optimal diagnosis and treatment options of human disease.

Selected experts were assigned to review all existing literature data, each on a specific aspect of human brucellosis treatment, and present them by analyzing the strength of evidence regarding each particular treatment option; a final session was held in order to set up the framework for the development of a common statement offering clinical perspectives for the 21st century. The statement was developed as a draft and re-circulated among the authors until a final form approved by all authors was reached. In developing the present therapeutic recommendations, guidelines of the Infectious Disease Society of America have been used (Table 1). The current recommendations are summarized in Table 2.

**Table 1:**
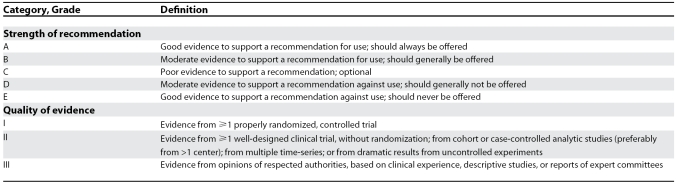
Infectious Diseases Society of America United States Public Health Service Grading System for Ranking Recommendations in Clinical Guidelines

**Table 2:**
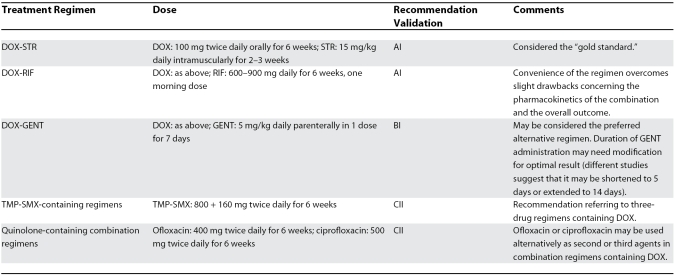
The recommendations of Ioannina on the Optimal Treatment of Brucellosis Without Serious Complications in Adults

A second position paper on the optimal diagnosis of the disease is currently under preparation.

## Efficacy of the WHO-Recommended Regimens

A meta-analysis performed in 1995 [12] evaluated the efficacy of the two WHO-recommended regimens and concluded that the efficacy of the doxycycline-streptomycin (DOX-STR) regimen was superior to that of the doxycycline-rifampicin (DOX-RIF) regimen, as already suggested by previous studies [13–15]. Even if subsequent randomized therapeutic trials were to be added to a future meta-analysis, the slight superiority of DOX-STR is obvious. The superior efficacy of the DOX-STR regimen was further supported by pharmacokinetic data (lower doxycycline serum levels induced by co-administration of rifampicin) [16,17] and resistance-related data: prolonged administration of rifampicin for brucellosis may increase the resistance of Mycobacterium tuberculosis to this compound in endemic areas. Moreover, experimental data suggested that the development of mycobacterial resistance to rifampicin may lead to development of resistance to other antimicrobials as well [18].

On the other hand, two recent studies have shown that the DOX-RIF regimen is preferred by both clinicians [19] and patients [20], even when they are aware of the relative superiority of the alternative regimen. The fact that DOX-RIF is an all-oral regimen may allow for better implementation in clinical practice in areas with less well-developed health infrastructure, because it eliminates the need for parenteral administration of streptomycin as part of the DOX-STR regimen. Furthermore, streptomycin shortage in numerous areas of the world may hamper the implementation of the DOX-STR regimen. Although evidence-based medicine would suggest that the DOX-STR regimen should always be offered, particularly in patients with complicated or serious forms of the disease, convenience, which can be translated into better adherence and overall success rates, forces the authors to suggest that DOX-RIF could be considered as an acceptable first-line regimen as well.

Regarding potential side-effects of these combinations, the authors suggest that the ototoxicity and nephrotoxicity of the aminoglycoside-containing regimens should be more thoroughly investigated in future randomized trials. The authors stress the need for inclusion of a tetracycline antibiotic in any therapeutic regimen for adult brucellosis. Various trials have been performed with combinations omitting such a compound, often reporting adequate results; yet understanding the pathophysiology of the disease renders the inclusion of tetracyclines imperative.

## Monotherapy

Monotherapy for brucellosis has generally been considered inadequate due to unacceptably high relapse rates. In older studies the use of tetracyclines as monotherapy was associated with varying relapse rates, ranging from 2% to 39% [21–26]. Nevertheless, these studies have major drawbacks: they were not randomized, they used doubtful diagnostic criteria, and the duration of treatment and adequacy of follow-up were widely varying. However, these criticisms do not apply to the excellent randomized comparative therapeutic trial performed by Montejo and colleagues [24]. This study showed that doxycycline monotherapy (100 mg twice daily for six weeks) is associated with relapse rates similar to the WHO-recommended regimen of DOX-RIF. The use of either trimethoprim-sulfamethoxazole (TMP-SMX) [24–31] or rifampicin [26,32] as monotherapy has also been reported in the past in adult brucellosis patients, with similarly varying results and similar reservations concerning the adequacy of the study design. A well-designed trial with TMP-SMX used for 45 days demonstrated a relapse rate of 46% [33].

Newer antibiotics such as ciprofloxacin and ceftriaxone have been tested as monotherapy in brucellosis in the past years, with disappointing results [34,35].

In the absence of definite data from randomized trials, the authors concluded, although not unanimously, that the available studies cannot convincingly support the use of monotherapy in human brucellosis. The authors however strongly recommend carefully designed prospective randomized clinical trials assessing the efficacy and safety of tetracycline monotherapy in patients with low relapse risk according to published data on relapse risk factors [9,10]. However, future single-regimen trials with other antibiotic classes are not justified.

## The Doxycycline-Gentamicin Regimen

Non-comparative studies on the efficacy of the combination of doxycycline and gentamicin (DOX-GENT) in brucellosis usually used shorter schedules of aminoglycosides than those of the standard regimens (i.e., 5–7 days for gentamicin) [12,36–38] with failure/ relapse rates ranging from 10% to 20% (the latter when doxycycline treatment duration was also shortened), which exceeded failure rates during the WHO-recommended treatment by about 5%. In the only randomized trial available of doxycycline administered for six weeks in combination with gentamicin for seven days, relapse rates were at least comparable to those reported for the WHO-recommended regimens [36].

The authors consider the DOX-GENT combination to be an adequate regimen for the treatment of human brucellosis that offers advantages over DOX-STR, given the wider availability of gentamicin and the sparing of streptomycin, a valuable anti-tuberculosis agent. However, the duration of gentamicin treatment should be further evaluated in future randomized control trials, as the authors were divided on whether gentamicin administration should be prolonged.

The aminoglycoside-containing combinations raise concerns about the applicability of a parenteral regimen in resource-deprived countries, because of the need for an adequate health infrastructure (for example walk-in clinics, staffed with experienced personnel, to deliver intramuscular injections daily) to eliminate the necessity of hospitalization, which would increase the overall treatment cost.

Although there are no brucellosis-specific data, the existing data support the feasibility of once-daily aminoglycoside dosing [39]. Interventions with gentamicin-encapsulated microspheres have shown promise in animal models and may prove useful alternative options in the future [40].

## Fluoroquinolone-Containing Regimens

Two recent reviews focused on fluoroquinolone-containing regimens for the treatment of brucellosis [41,42]. Both reviews, although using different approaches, conclude that combinations including fluoroquinolones can be acceptable alternatives, but do not recommend them as first-line options for human brucellosis treatment. According to the first review, the few proper randomized trials existing on the subject do not indicate the superiority or even non-inferiority of fluoroquinolone-containing regimens. The second review reports a cumulative response rate of above 85%, which is an adequate response; however, the currently higher cost of fluoroquinolone-containing regimens and the risk of enhancing the development of overall fluoroquinolone resistance in the community argue against wide use of fluoroquinolones for human brucellosis. The authors feel that in the future, if older fluoroquinolones become less expensive, further properly designed randomized trials may explore their potential in combined brucellosis treatment. There are no data, apart from sporadic case reports, on the efficacy of the newer fluoroquinolones in brucellosis, but in view of their unique efficacy against respiratory pathogens the authors do not recommend their routine use in brucellosis. Therefore, these antibiotics should be administered only in the context of properly designed prospective clinical trials.

## TMP-SMX-Containing Regimens

TMP-SMX has been a popular choice, and was included in various combination regimens around the world, due to its significantly lower cost (compared to other antimicrobials included in treatment regimens for brucellosis), which rendered it the most cost-effective drug against brucellosis in certain developing world counties. Data on its interaction with rifampicin suggest a potential beneficial increase in the latter's serum levels [43], but a synergistic effect has not been proven [44,45]. TMP-SMX combination with rifampicin has proven successful in many reports of pediatric brucellosis. In adults, a recent randomized study suggested an adequate (but not superior) response rate comparable to the WHO-recommended regimens, although the treatment duration was extended [46]. Past studies have produced contradicting results, with relapse rates ranging from 0% to 30% (TMP-SMX combinations with tetracycline) [25,47].

TMP-SMX has been extensively used in triple combinations in some countries. The overall reported response rate was above 90% for the various TMP-SMX-containing combinations, although the absence of doxycycline from the regimen was related to an unacceptably high percentage of treatment failures (M. Bosilkovski, unpublished data). These data were derived from clinical studies which included patients with severely complicated disease; therefore extrapolation of these conclusions to patients with uncomplicated brucellosis is not easy. At present, the routine use of a triple regimen containing TMP-SMX cannot be advocated. However, the authors suggest that, depending on the regional overall failure rates of the WHO-recommended regimens, TMP-SMX may be used as an additional third (and only third) antibiotic.

One important issue regarding the use of TMP-SMX in brucellosis treatment is the development of resistance in B. melitensis. Previous studies have suggested that TMP-SMX use has been associated with increasing B. melitensis resistance rates, sometimes reaching 62% [48,49]. Resistance rates may vary over time, reflecting the antibiotics' overall use for brucellosis [50]. If a DOX/TMP-SMX combination is to be considered for a future clinical trial, the problem of resistance to TMP-SMX should be seriously taken into account. (The well-known dissociation between in vitro activity and in vivo activity—i.e., therapeutic failure in vivo—in brucellosis refers only to drugs with in vitro activity and not in vivo, but not vice versa. This means that if in vitro activity is absent, for example as with most beta-lactams, clinical failure is guaranteed.) In future studies, isolated strains should be carefully studied for resistance to TMP-SMX and the other agents active against Brucella, and the relation of overall antibiotic use to the development of resistance should be analyzed in every region.

## Other Tetracyclines and Related Compounds

A number of Italian studies have investigated the use of minocycline instead of doxycycline in the treatment of brucellosis. Early studies from the 1980s used minocycline either as monotherapy or in combination with intravenous rifampicin for short periods (2–4 weeks); both antimicrobials were delivered initially intravenously and subsequently orally. All of the above studies reported very low relapse rates, and were summarized in a recent article [51]. However, these data should be interpreted cautiously because the studies were retrospective and not comparative. The potential superiority of minocycline over doxycycline could be attributed to its increased bioavailability and the increased area under the curve of free minocycline compared to free doxycycline, as well as minocycline's greater lipophilicity, which allows for better tissue penetration [52]. The authors stress that, given wide anecdotal reports of lower adherence to minocycline treatment (compared to doxycycline), a combination including minocycline would have to prove far superior compared to doxycycline to merit further consideration.

Regarding oxytetracycline, at present it might be considered as a less convenient, in terms of multiple daily doses, but adequate alternative to doxycycline.

Tigecycline is the first glycylcycline antibiotic, a new member of the tetracycline family with chemical structure similar to that of minocycline. The drug has the ability to overcome the two major resistance mechanisms of tetracycline—drug-specific efflux pump acquisition and ribosomal protection—and is active against many gram-positive and gram-negative organisms [53]. The authors believe that the potential role of this new compound in the treatment of brucellosis may be hampered by problems such as the cost and the need for parenteral administration; a future clinical trial using this compound would need to show substantial superiority regarding efficacy and treatment duration in order to overcome these reservations. Future studies should also explore a possible role of tigecycline in the therapy of serious clinical forms of the disease requiring intravenous antibiotics.

## Antibiotics Not Suitable for Brucellosis Treatment

Numerous other antibiotic classes have been clinically tested in the treatment of brucellosis. Azithromycin has been shown to be inadequate in a small but well-randomized trial [54]. Data from literature from Russia suggest a potential role for meropenem [55], yet the cost and importance of this agent for the treatment of more serious infections lead the authors to advocate against further clinical trials with this agent. However, its use might be considered in an individual hospitalized case as an alternative or as salvage therapy.

## Special Situations

Treatment of brucellar spondylitis varies widely; one recent attempt at a meta-analysis of the existing data concluded that what matters for the outcome is the duration of treatment, and not the specific (recommended) regimen used [56]. However, many of the authors believe that some principles do apply in the treatment of brucellar spondylodiscitis: aminoglycoside-containing regimens may be superior to rifampicin-containing ones [57]; magnetic resonance imaging (MRI) of the spine should always be performed when there is clinical suspicion of spinal involvement irrespective of the rachideal level potentially affected; and the spine (especially if there is cervical involvement) should be immobilized in order to avoid devastating neurological complications. Although the previously mentioned meta-analysis did not conclude that a streptomycin-containing regimen may be superior, the authors suggest that the outcome of spondylitis may potentially be improved when such a regimen is used. According to certain authors this approach may be successful (in patients who have spondylitis only and not associated paravertebral or epidural abscess) even if duration of treatment is limited to the six weeks' therapeutic course used in uncomplicated brucellosis [58]. At present though, the available data support a longer duration of treatment of not less than three months [56,59]. Another important subject for future properly designed clinical trials would be the utility of MRI in the follow-up of these patients, since conflicting data exist on the subject, i.e., should treatment be continued until resolution of spinal MRI findings or not.

Neurobrucellosis is a term encompassing a wide spectrum of central nervous system clinical manifestations. Although central nervous system involvement is probably more common than originally considered, the paucity of therapeutic data preclude any recommendations to be offered for the time being.

Childhood brucellosis will be discussed in a separate statement currently being developed.

There are no randomized trials for brucellosis in pregnancy. The most extended series support the use of TMP-SMX alone or in combination with rifampicin [60]. The potential for adverse effects on the fetus from antimicrobials makes brucellosis in pregnancy a key situation where monotherapy trials are needed. For example, patients without risk factors for relapse or those without focal disease may be treated with rifampicin monotherapy (arguably the safer of all available antibiotics for brucellosis in pregnancy) until delivery. The advantage of minimizing the risks for fetal health is counterbalanced by the disadvantage of higher relapse rates; however, in case of postpartum relapse, the patient can be treated with a standard regimen.

Brucellar endocarditis is a notorious complication with high mortality that has been reported fewer than 200 times in the literature; therefore no randomized data are available. Although a report has suggested that certain factors may allow for conservative treatment [61], the selection of antimicrobials remains empirical; in the majority of reported cases surgical intervention was necessary. The authors suggest that through a large, international database of brucellosis cases more endocarditis cases could be recorded, and the risk factors, pathophysiology, natural history, and clinical characteristics of this complication could be better characterized. This information would enable the design of an effective therapeutic approach.

## Developing a Common Language

One of the major obstacles in the clinical research on brucellosis is the absence of properly designed and randomized therapeutic trials. Standards applying to typical bacterial infections cannot be easily implemented in brucellosis research; the concept of microbiological cure is vague in brucellosis. Moreover, the concept of relapse is in itself vague as well: is relapse a clinical failure? And if so, what is the required period of follow-up after treatment completion? The pathogen's virulence may also vary in different regions of the world: this variation is not only because of different Brucella strains (B. melitensis versus B. abortus, etc.), but also to different B. melitensis biovars that are endemic in each country. Important issues that should be addressed in the future include the optimization of treatment adherence, the monitoring for potential adverse effects, the importance of expert consultation in complicated disease, and the importance of a good relationship between patients and physicians, which is essential for a better prognosis, especially in cases of chronic brucellosis.

Regarding the nature of chronic disease: All attempts to categorize the disease have been hampered by the lack of agreement on terminology and the arbitrary nature of the designations. Nevertheless, many published works divide patients into acute versus chronic disease, often based on clinical complaints alone, or on some arbitrary number of days of symptoms. Although this classification does not usually affect the regimens used in various antibiotic treatment studies, the development of a common terminology is of paramount importance in order to better design effectively large, international, multicenter clinical studies.

## Abbreviations

DOX-GENT: doxycycline-gentamicin
DOX-RIF: doxycycline-rifampicin
DOX-STR: doxycycline-streptomycin
MRI: magnetic resonance imaging
TMP-SMX: trimethoprim-sulfamethoxazole
WHO: World Health Organization
